# Structural basis for inhibition of the RNA-dependent RNA polymerase from SARS-CoV-2 by remdesivir

**DOI:** 10.1126/science.abc1560

**Published:** 2020-05-01

**Authors:** Wanchao Yin, Chunyou Mao, Xiaodong Luan, Dan-Dan Shen, Qingya Shen, Haixia Su, Xiaoxi Wang, Fulai Zhou, Wenfeng Zhao, Minqi Gao, Shenghai Chang, Yuan-Chao Xie, Guanghui Tian, He-Wei Jiang, Sheng-Ce Tao, Jingshan Shen, Yi Jiang, Hualiang Jiang, Yechun Xu, Shuyang Zhang, Yan Zhang, H. Eric Xu

**Affiliations:** 1 The CAS Key Laboratory of Receptor Research, Shanghai Institute of Materia Medica, Chinese Academy of Sciences, Shanghai 201203, China.; 2 Department of Biophysics, and Department of Pathology of Sir Run Run Shaw Hospital, Zhejiang University School of Medicine, Hangzhou 310058, China.; 3 School of Medicine, Tsinghua University, Haidian District, Beijing 100084, China.; 4 Department of Cardiology, Peking Union Medical College Hospital, Peking Union Medical College and Chinese Academy of Medical Sciences, Beijing 100730, China.; 5 Tsinghua-Peking Center for Life Sciences, Tsinghua University, Beijing 100084, China.; 6 University of Chinese Academy of Sciences, Beijing 100049, China.; 7 WuxiBiortus Biosciences Co. Ltd., Jiangyin 214437, China.; 8 Center of Cryo-Electron Microscopy, Zhejiang University School of Medicine, Hangzhou 310058, China.; 9 Center of Diagnostic Electron Microscopy, Sir Run Run Shaw Hospital, Zhejiang University School of Medicine, Hangzhou 310058, China.; 10 Shanghai Center for Systems Biomedicine, Key Laboratory of Systems Biomedicine (Ministry of Education), Shanghai Jiao Tong University, Shanghai 200240, China.; 11 Key Laboratory of Immunity and Inflammatory Diseases of Zhejiang Province, Hangzhou 310058, China.

## Abstract

The pandemic of coronavirus disease 2019 (COVID-19), caused by severe acute respiratory syndrome coronavirus 2 (SARS-CoV-2), has become a global crisis. Replication of SARS-CoV-2 requires the viral RNA-dependent RNA polymerase (RdRp) enzyme, a target of the antiviral drug remdesivir. Here we report the cryo–electron microscopy structure of the SARS-CoV-2 RdRp, both in the apo form at 2.8-angstrom resolution and in complex with a 50-base template-primer RNA and remdesivir at 2.5-angstrom resolution. The complex structure reveals that the partial double-stranded RNA template is inserted into the central channel of the RdRp, where remdesivir is covalently incorporated into the primer strand at the first replicated base pair, and terminates chain elongation. Our structures provide insights into the mechanism of viral RNA replication and a rational template for drug design to combat the viral infection.

## A wrench in the works of COVID-19

Understanding the inner workings of the virus that causes coronavirus disease 2019 (COVID-19) may help us to disrupt it. Yin *et al.* focused on the viral polymerase essential for replicating viral RNA. They determined a structure of the polymerase bound to RNA and to the drug remdesivir. Remdesivir mimics an RNA nucleotide building block and is covalently linked to the replicating RNA, which blocks further synthesis of RNA. The structure provides a template for designing improved therapeutics against the viral polymerase.

*Science*, this issue p. 1499

## Teaser

Cryo-EM structures show how the drug remdesivir binds to the RNA polymerase to block RNA elongation.

The coronavirus disease 2019 (COVID-19) pandemic that has arisen from widespread severe acute respiratory syndrome coronavirus 2 (SARS-CoV-2) infection has become a humanitarian crisis, with more than 1.5 million infections and 87,000 deaths reported on 8 April 2020 (*1*, *2*). These numbers have increased rapidly to more than 2.99 million infections and 207,000 deaths as of 27 April of 2020 (*2*). SARS-CoV-2 is closely related to severe acute respiratory syndrome coronavirus (SARS-CoV) and several members of the betacoronavirus family, including bat and pangolin coronaviruses (*3*–*5*). Compared with the binding behavior of other coronaviruses, however, the spike protein of SARS-CoV-2 has a stronger binding affinity for the host receptor (*6*–*10*), which may explain why SARS-CoV-2 has a much higher incidence of human-to-human transmission, resulting in infections throughout the world.

SARS-CoV-2 is a positive-strand RNA virus. Its replication is mediated by a multisubunit replication-and-transcription complex of viral nonstructural proteins (nsps) (*11*). The core component of this complex is the catalytic subunit (nsp12) of an RNA-dependent RNA polymerase (RdRp) (*12*, *13*). By itself, nsp12 has little activity and its functions require accessory factors, including nsp7 and nsp8 (*14*, *15*), that increase RdRp template binding and processivity. RdRp is also proposed to be the target of a class of antiviral drugs that are nucleotide analogs; this category includes remdesivir (*16*–*18*), which is a prodrug that is converted to the active drug in the triphosphate form [remdesivir triphosphate (RTP)] within cells (*19*). As such, RdRp has been the subject of intensive structural biology efforts. The structures of nsp7, nsp8, and the complex of nsp12-nsp7-nsp8 have been determined (*15*, *20*–*23*), providing the overall architecture of the RdRp complex. However, the drug discovery effort is hampered because the structures of the SARS-CoV-2 RdRp in complex with an RNA template and with nucleotide inhibitors are not known. In this study, we determined two cryo–electron microscopy (cryo-EM) structures of the SARS-CoV-2 RdRp complex: one in the apo form and the other in a complex with a template-primer RNA and the antiviral drug remdesivir.

**Fig. 1:**
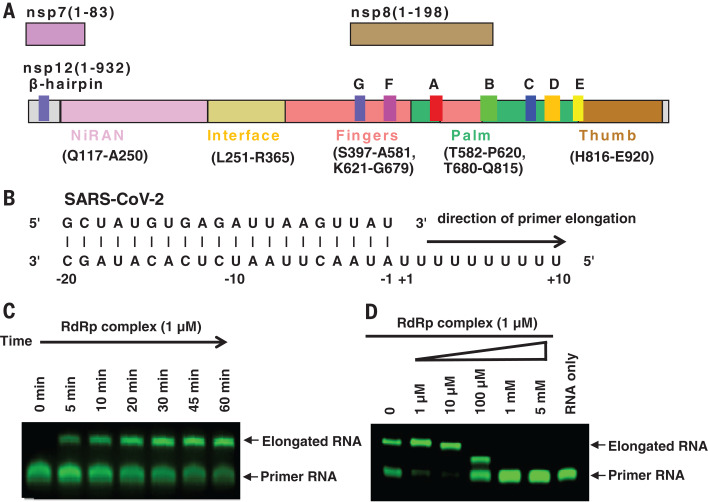
Assembly of an active nsp12-nsp7-nsp8 RdRp complex and its inhibition by remdesivir. (**A**) Schematic diagram for the components of the RdRp complex, containing nsp12, nsp7, and nsp8. The polymerase motif (A to G) and the β hairpin specific to SARS-CoV-2 are highlighted. (**B**) Sequence of the RNA duplex with a 5′ U_10_ overhang as a template for primer extension and RdRp-RNA complex assembly. (**C**) The recombinant RdRp complex shows polymerase activity in vitro. The primer strand is labeled with fluorescence at the 5′ end. (**D**) Elongation of the partial RNA duplex by the purified RdRp complex and its inhibition by RTP.

For cryo-EM studies, we coexpressed nsp12 with nsp7 and nsp8 to form the core RdRp complex in insect cells (Fig. 1A and fig. S1, A to D). The stoichiometric amount of nsp7 and nsp8 appeared to be less than that of nsp12, and thus additional bacterially expressed nsp7 and nsp8 were supplemented before the final purification step to improve the yield of heterotrimeric complex. The purified nsp12 alone showed little activity in binding to a 50-base partial double-stranded template-primer RNA (Fig. 1B and fig. S1E), which is similar to the SARS-CoV nsp12 (*14*). The presence of nsp7 and nsp8 markedly increased nsp12 binding to the template-primer RNA (fig. S1E). The nsp12-nsp7-nsp8 complex also showed RNA polymerization activity on a poly-U template upon addition of adenosine triphosphate (ATP) (Fig. 1, B and C). This RNA polymerization activity was effectively inhibited by the addition of RTP (Fig. 1D). Even in the presence of 10 mM ATP, which is within the range of physiological concentrations of ATP, 1 mM RTP completely inhibited RdRp polymerization activity. In addition, 100 µM RTP completely blocked the full extension but allowed partial extension of the primer RNA (Fig. 1D), consistent with a delayed chain termination mechanism (*24*). However, this mechanism is dependent on low RTP concentrations and low RTP/ATP ratios. By contrast, 5 mM remdesivir (as a prodrug) had no inhibitory effect on the polymerization activity of the purified enzyme (fig. S1F), nor did remdesivir in its monophosphate form (RMP) (fig. S1G).

The purified RdRp complex is relatively thermostable, with a melting temperature of 53°C (fig. S1H). Negative-stain EM visualization of the nsp12-nsp7-nsp8 complex displayed monodispersed particles with excellent homogeneity (fig. S1I). For the apo nsp12-nsp7-nsp8 complex, we vitrified the sample in the presence of the detergent DDM. The initial attempt at image processing revealed that the particles are preferentially oriented (fig. S2A). Therefore, we collected >7400 micrograph movies of more than 5.7 million particle projections to increase the number of projections from the nonpreferential orientation. Of these, 81,494 particles were used to yield a density map of 2.8-Å resolution (fig. S2, B and E). Cryo-EM studies of the nsp12-nsp7-nsp8 complex bound with the template-primer RNA and RTP (termed the template-RTP RdRp complex) faced two challenges (fig. S3). First, most particles were adsorbed to cryo-EM grid bars rather than staying in the vitreous ice. Second, the RNA duplex was dissociated from the template-RTP RdRp complex, likely owing to conditions of cryo-EM specimen preparation. Eventually, we prepared the cryo-EM specimen of the template-RTP RdRp complex at 15 mg/ml, which is much higher than the normal concentrations used for EM studies of soluble protein complexes. The high concentration of the complex has a mass action effect to stabilize the RNA-protein complex and has an excess amount of the complex to escape the absorption of cryo-EM grid bars to enter the vitreous ice (fig. S3). We collected 2886 micrograph movies, which yielded a 2.5-Å resolution structure using 130,386 particle projections. Because of the relatively high resolution of our structure, the EM map is clear for key structural features across the complex (fig. S4, A to F).

**Fig. 2:**
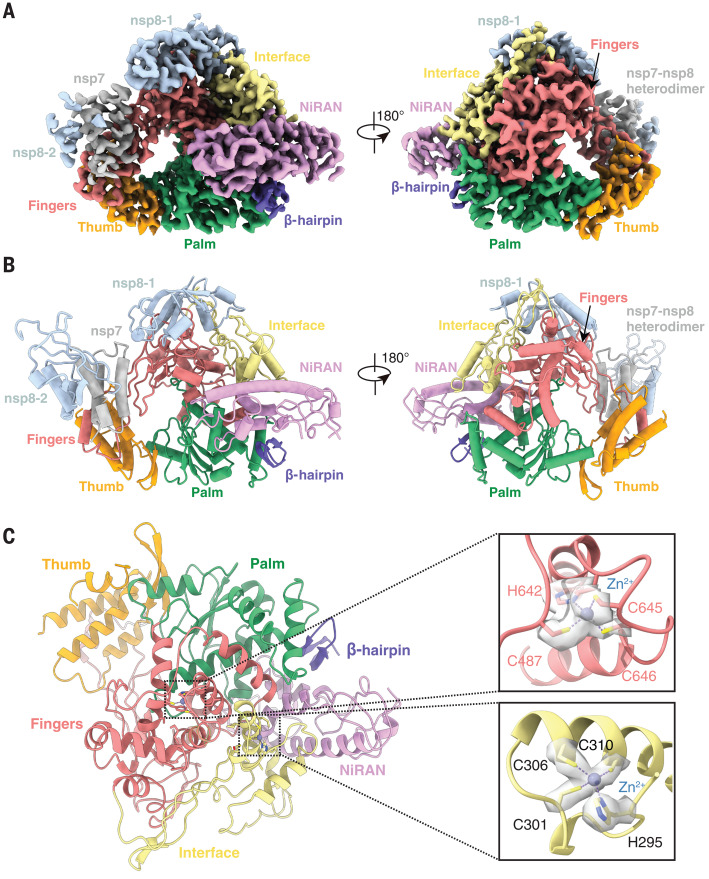
Cryo-EM structure of the apo nsp12-nsp-7-nsp8 RdRp complex. (**A** and **B**) Two views of the cryo-EM map (A) and structure (B) of the apo nsp12-nsp7-nsp8 complex. The color scheme is according to Fig. 1A and is used throughout the figures. (**C**) The conserved zinc binding motifs are highlighted in the apo structure rendered in ribbon. The coordinate details of the zinc-binding residues are shown in stick representation, with the EM map in gray surface representation.

The structure of the apo RdRp complex contains one nsp12, one nsp7, and two nsp8 proteins, with an overall arrangement resembling those seen in SARS-CoV and the recently solved structure of SARS-CoV-2 (*15*, *23*) (Fig. 2, A and B). Our structure, which differs from the SARS-CoV RdRp structure but is similar to the recent SARS-CoV-2 RdRp structure, reveals that nsp12 also contains an N-terminal β hairpin (residues 31 to 50) and an extended nidovirus RdRp-associated nucleotidyl-transferase domain (NiRAN; residues 115 to 250) (*24*) with seven helices and three β strands (*15*, *23*). After the NiRAN domain is an interface domain (residues 251 to 365), composed of three helices and five β strands, that is connected to the RdRp domain (residues 366 to 920) (Figs. 1A and 2B). The nsp12 RdRp domain displays the canonical cupped right-handed configuration (*25*), with the finger subdomain (residues 397 to 581 and 621 to 679) forming a closed circle with the thumb subdomain (residues 819 to 920) (Fig. 2, A and B). The closed conformation is stabilized by the binding of nsp7 and nsp8, with one nsp8 molecule sitting atop the finger subdomain and interacting with the interface domain. The closed conformation of nsp12 is further stabilized by the nsp7-nsp8 heterodimer, which is packed against the thumb-finger interface (Fig. 2, A and B). In addition, we were able to assign two zinc ions in the conserved metal binding motifs composed of H295-C301-C306-C310 and C487-H642-C645-C646 (Fig. 2C), which are also observed in the SARS-CoV RdRp structure (*15*). These zinc ions likely function as conserved structural components in maintaining the integrity of the RdRp architecture.

**Fig. 3:**
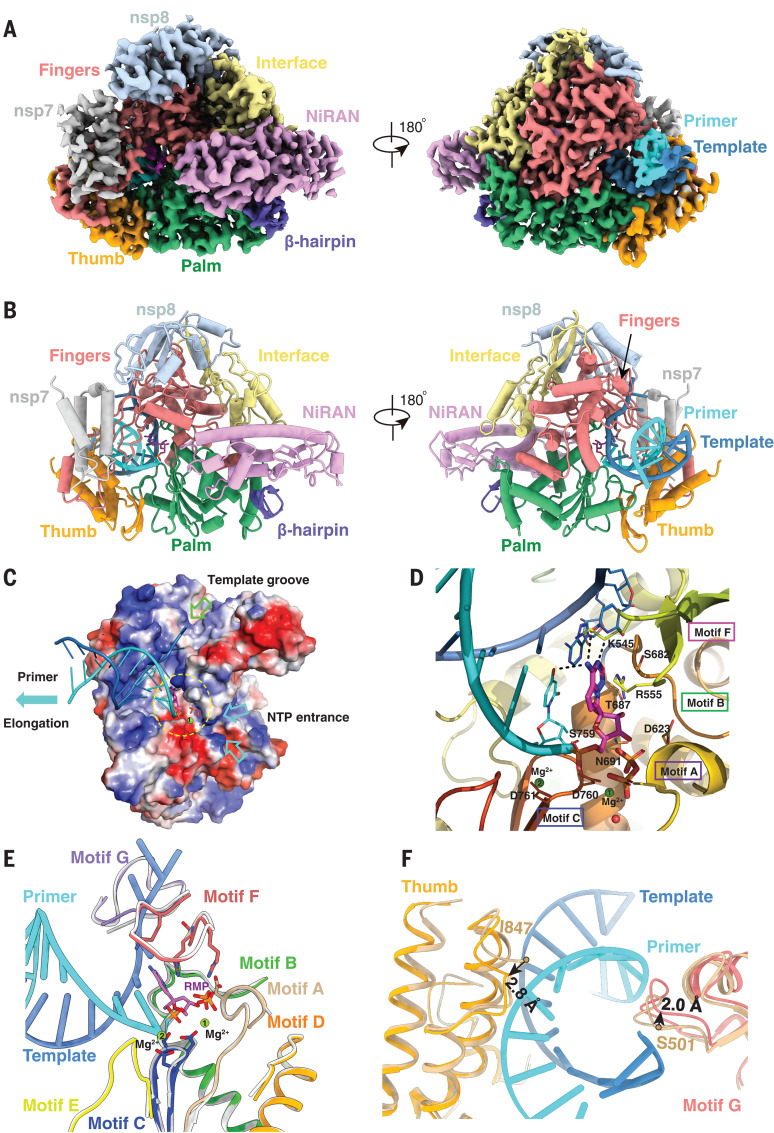
Cryo-EM structure of the remdesivir- and RNA-bound RdRp complex. (**A** and **B**) Two views of the cryo-EM map (A) and structure (B) of nsp12-nsp7-nsp8 in complex with template-primer RNA and remdesivir. (**C**) Surface view of the RdRp active site with the electrostatic potential from red (negative) to blue (positive). For clarity, residues 410 to 442 and 834 to 919 of nsp12 and nsp8 are excluded from the figure. The covalently bound remdesivir in the monophosphate form and the product, pyrophosphate, are shown. The active site is emphasized with a yellow dashed circle. The template groove, the entrance for nucleotide triphosphate (NTP), and the elongation direction are annotated with different-colored arrows. (**D**) Close-up view of the RdRp active site, showing the covalently bound RMP, pyrophosphate, and magnesium ions. Key residues and bases that interact with remdesivir are shown. (**E** and **F**) Superposition of the conserved RdRp motifs (A to G) of the RNA-bound complex with the apo structure (colored in gray), with a close-up view at the active site (E) and at the exit of the template and primer strand (F).

The structure of the template-RTP RdRp complex contains one nsp12, one nsp7, and one nsp8 (Fig. 3, A and B). The second nsp8 was largely invisible in the EM map of the template-RTP complex (fig. S4C); therefore, it was not included in the final model. In addition, the template-RTP RdRp structure contains 14-base RNA in the template strand, 11-base RNA in the primer strand, and the inhibitor RMP (Fig. 3, C and D), which is covalently linked to the primer strand, as well as a pyrophosphate and two magnesium ions that may serve as catalytic ions near the active site (Fig. 3D and fig. S4, D to F) (*26*).

**Fig. 4:**
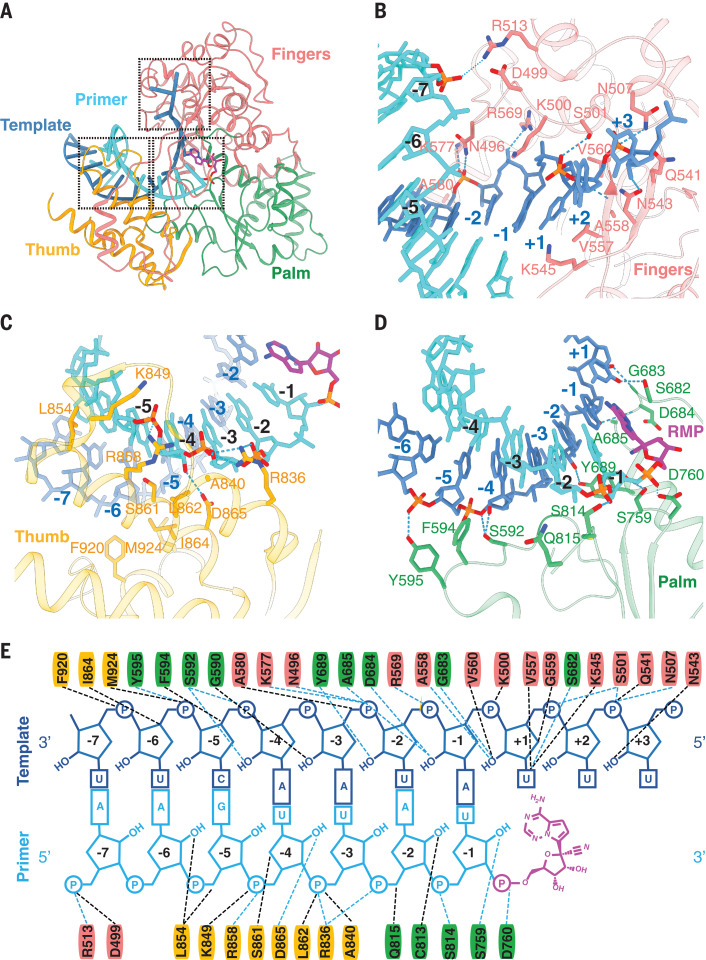
RNA recognition by the RdRp complex. (**A** to **D**) Protein-RNA interactions in the RNA- and remdesivir-bound RdRp complex. (**E**) Diagram of detailed RNA interactions with RdRp. Single-letter abbreviations for the amino acid residues are as follows: A, Ala; C, Cys; D, Asp; E, Glu; F, Phe; G, Gly; H, His; I, Ile; K, Lys; L, Leu; M, Met; N, Asn; P, Pro; Q, Gln; R, Arg; S, Ser; T, Thr; V, Val; W, Trp; and Y, Tyr.

The overall structure of the template-RTP RdRp complex is similar to the apo RdRp structure, with nsp12 in a closed conformation (Figs. 2A and 3A). The double-stranded RNA helix, formed by 11 base pairs from the template-primer RNA (Figs. 3C and 4, A to E), is held by the finger-palm-thumb subdomains. Extensive protein-RNA interactions are observed between the template-primer RNA and nsp12, with a total of 41 residues from nsp12 directly participating in RNA binding (within 4.0 Å of RNA, 26 residues to the template strand and 15 residues to the primer strand; Fig. 4E). Surprisingly, no RNA interactions are mediated by nsp7 or nsp8, although these two proteins are required for RNA binding by RdRp. Most protein-RNA interactions involve the RNA phosphate-ribose backbones, with many interactions directly to 2′-OH groups (Fig. 4E), thus providing a basis to distinguish RNA from DNA. There are no contacts from nsp12 to any base pairs of the template-primer RNA, suggesting a sequence-independent binding of RNA by RdRp. This is consistent with the fact that no specific sequence is required for the enzymatic activity of RdRp at the elongation step.

At the 3′ end of the primer strand is RMP (Figs. 3D and 4, D and E, and fig. S4, E and F), which is covalently incorporated into the primer strand at the +1 position (Fig. 4E). Additional nucleotides at the +2 and +3 positions of the template strand interact with residues from the back of finger subdomain (Fig. 4, A and B). Despite the presence of excess RTP in complex assembly, only a single RMP is assembled into the primer strand, as observed in the structure. Consistent with the data from Fig. 1D, the primer extension is immediately terminated when RTP concentration is high and ATP/RTP ratio is low. Thus, remdesivir, like many nucleotide analog prodrugs, inhibits viral RdRp activity through nonobligate RNA chain termination, a mechanism that requires conversion of the parent drug to the active triphosphate form (*27*, *28*).

The RMP position is at the center of the catalytic active site (Fig. 3D). As an adenosine monophosphate analog, RMP forms base-stacking interactions with an upstream base from the primer strand and two hydrogen bonds with the uridine base from the template strand (Fig. 3D and fig. S5). In addition, RMP forms interactions with side chains from K545 and R555. Near the bound RMP are two magnesium ions and a pyrophosphate. Both magnesium ions interact with the phosphate diester backbone and are part of the catalytic active site. The pyrophosphate is at the gate of the nucleotide entry channel to the active site and may block the entry of nucleotide triphosphate to the active side (Fig. 3, C and D).

The catalytic active site of the nsp12 RdRp consists of seven conserved motifs (A to G; Figs. 1A and 3E and fig. S6). Motifs A, B, C, and D are from the palm subdomain, with an SDD sequence (residues 759 to 761) in motif C forming the catalytic active center (Fig. 3D). Both D760 and D761 are involved in coordination of the two magnesium ions at the catalytic center. Motifs F and G are located within the finger subdomain; they interact with the template strand RNA and direct this strand into the active site (Fig. 3E). Motif F also interacts with the primer strand RNA, with the side chains of K545 and R555 contacting the +1 base (Fig. 3D) and thus stabilizing the incoming nucleotide in the correct position for catalysis. The orientation of template-primer RNA in the active site is similar to the orientation of template-primer RNA in the poliovirus RdRp elongation complex (*29*) and the hepatitis C virus NS5B RdRp inhibitor complex (*30*) (fig. S7). The residues involved in RNA binding and those that constitute the catalytic active site are highly conserved (*31*, *32*), highlighting the conserved mechanism of genome replication by RdRp in these diverse RNA viruses and suggesting that it may be possible to develop broad-spectrum antiviral inhibitors such as remdesivir (*18*) and galidesivir (BCX4430) (*33*).

Structural comparison reveals several notable differences between the apo and complex structures (Fig. 3, E and F, and fig. S8, A and B). First, nsp7 moves toward the RdRp core by 1.5 Å (as measured by nsp7 residue F49; fig. S8, A and B), leading to a rearrangement of the interface, which results in weaker association of the second nsp8 in the complex. Second, the loop that connects the first and second helices of the thumb subdomain moves outward by 2.8 Å (as measured by nsp12 residue I847) to accommodate the binding of the double-stranded RNA helix (Fig. 3F). Third, motif G residues K500 and S501 also move outward by 2.0 Å to accommodate the binding of the template-strand RNA. Outside of these changes, the apo nsp12 and the RNA complex nsp12 are very similar, with a root mean square deviation of 0.52 Å for all Cα atoms across the whole protein. In particular, the structural elements that make up the catalytic active site can be precisely superimposed (Fig. 3E), which suggests that the SARS-CoV-2 RdRp is a relatively stable enzyme that is ready to function as a replicase upon RNA template binding. Viral RdRp is a highly processive enzyme with a replication rate of up to 100 nucleotides per second (*34*). No substantial conformational changes between the apo and active enzyme structures are consistent with the high processivity of the viral RNA polymerase, which does not need to consume additional energy for conformational changes in the active site during the replication cycle.

Besides remdesivir, several nucleotide analog drugs—including favipiravir, ribavirin, galidesivir, and EIDD-2801—efficiently inhibit SARS-CoV-2 replication in cell-based assays (*35*, *36*). Like remdesivir, these nucleotide analogs are proposed to inhibit the viral RdRp through nonobligate RNA chain termination, a mechanism that requires conversion of the parent compound to the triphosphate active form (*33*). The structure of the template-RTP RdRp complex provides a useful model to rationalize how these drugs inhibit the activity of SARS-CoV-2 RdRp (fig. S8C). In particular, EIDD-2801 has been shown to be 3 to 10 times as potent as remdesivir in blocking SARS-CoV-2 replication (*36*). The N4 hydroxyl group off the cytidine ring forms an extra hydrogen bond with the side chain of K545, and the cytidine base also forms an extra hydrogen bond with the guanine base from the template strand. These two extra hydrogen bonds may explain the apparent higher potency of EIDD-2801 in inhibiting SARS-CoV-2 replication.

The COVID-19 pandemic has inflicted emotional pain and economic burden across the globe. Enzymes that are vital for the viral life cycle are suitable antiviral drug targets because they differ from host proteins. Among viral enzymes, RdRp is the primary target of many existing nucleotide drugs. In this paper, we report the structures of the SARS-CoV-2 RdRp complex in the apo form as well as in complex with a template-primer RNA and the active form of remdesivir. The structures reveal how the template-primer RNA is recognized by the enzyme and how chain elongation is inhibited by remdesivir. Structure comparison and sequence alignment suggest that the mode of substrate RNA recognition and remdesivir inhibition of RdRp is highly conserved in diverse RNA viruses, providing a foundation for designing broad-spectrum antiviral drugs based on nucleotide analogs. Moreover, our structures provide a solid template for modeling and modifying the existing nucleotide drugs, including the highly potent EIDD-2801. Together, these observations provide a rational basis to design even more potent inhibitors to combat SARS-CoV-2 infection.

## Notes

**Correction (25 June 2020):** After the initial online publication of the paper, the structures for Protein Data Bank (PDB) IDs 7BV1 and 7BV2 were updated, with corrections in the C-terminal fragment of nsp12 (residues 911 to 929) and removal of the third magnesium ion. As such, Figs. 3E and 4 have also been updated, along with the corresponding text.

## Supplementary Materials

science.sciencemag.org/content/368/6498/1499/suppl/DC1 Figs. S1 to S8 Table S1 References (*37*–*46*)

## Appendix

View/request a protocol for this paper from *Bio-protocol*.
